# Best Practices to Advance Health Literacy in a Wisconsin Adult Literacy Coalition

**DOI:** 10.3928/24748307-20190405-01

**Published:** 2019-10-03

**Authors:** Michele Erikson, Paul D. Smith, Steven W. Sparks

## Abstract

**Background:**

Wisconsin Literacy Incorporated is a statewide adult and family literacy coalition representing 75 community-based literacy organizations. This coalition focuses its work in four areas: (1) building capacity of its member agencies, (2) advocating for literacy, (3) preparing adults in worker readiness and career pathways, and (4) improving how health information is communicated through its division, Wisconsin Health Literacy (WHL).

**Brief Description of Activity:**

This article outlines how an adult basic education coalition successfully developed a statewide health literacy coalition that later transformed into a division of Wisconsin Literacy through a three-phase approach that included: (1) raising awareness and educating health care and literacy providers about health literacy, (2) implementing health literacy interventions through various grant funding, and (3) disseminating and sharing findings from such health literacy interventions to a broad audience.

**Implementation:**

Beginning its awareness work in 2003 with help from a physician champion, WHL implemented best practices within each of the three areas of approach. After being implemented, the initial volunteer-based health literacy coalition was transformed into a division of Wisconsin Literacy Incorporated.

**Results:**

The division now includes a full-time director, its own website, funding for community interventions, and a business plan for fee-for-service efforts with health care providers, public health agencies, and other stakeholders. Key relationships with Wisconsin health care organizations provided WHL venues to present about health literacy throughout the state and beyond. Wisconsin Literacy Incorporated helped secure a solid infrastructure by hosting two planning retreats and implementing both strategic and business plans for the newly formed division. Offering fee-for-service health literacy training to health care providers brought in new revenue to sustain the division and obtain buy-in from health care agencies on the importance of health literate organizations. Finally, participating in key partner coalitions and Communities of Practice allowed WHL to share experiences and best practices with a national audience.

**Lessons Learned:**

The importance of intentionally raising awareness in strategic health care settings across the state fostered many partnerships. Investing in a strategic planning retreat and a business plan guided the division's success. Delivering educational workshops in community-based literacy organizations and trusted settings where adults regularly go to learn was key to successful implementation. **[*HLRP: Health Literacy Research and Practice*. 2019;3(Suppl.):S8–S14.]**

**Plain Language Summary:**

Wisconsin Literacy Incorporated is an adult basic education coalition that built a health literacy division over several years. Using best practices such as raising awareness, implementing pilot projects, and broadly sharing its experience, the Wisconsin Health Literacy division advanced the understanding and commitment to health literacy in and beyond Wisconsin.

This article reports on how Wisconsin Literacy Incorporated became involved in health literacy efforts and the processes and best practices that were developed to build a health literacy division within its statewide coalition of adult literacy agencies. The article describes the development of Wisconsin Health Literacy (WHL), a division of Wisconsin Literacy Incorporated, and shares how incorporating health literacy into a statewide literacy coalition can positively affect a state's understanding and commitment to enhancing health literacy.

Formed in 1985, Wisconsin Literacy Incorporated provides 75 local literacy agencies with resources, professional development, training, and advocacy to build their capacity to serve adults and families looking to improve their literacy skills or learn English as a second language. Although the organization had existed as a 501(c)(3) nonprofit entity since 1985, it was an all-volunteer coalition and lacked the resources, especially paid staff, needed for programing in health literacy. Seed funding was secured in 2003 to open an office with a part-time director, and doors began to open for the coalition to expand its reach and scope of activity. This also coincided with a time when the field of health literacy was gaining national attention among health care providers, and to a lesser extent, adult education providers. New conversations were occurring in health care organizations, and seminal reports such as the Institute of Medicine's *Health Literacy: A Prescription to End Confusion* (Kindig, Panzer, & Nielsen-Bohlman, 2004) were being shared, but the extent to which literacy rates were affecting communities, labor markets, and educational attainment levels was not yet a big part of the conversation. During this time, a physician in Wisconsin became interested in issues of literacy and health literacy and their impact on health, and he eventually became a health literacy champion.

In 2003, the National Center for Education Statistics released the National Assessment of Adult Literacy (NAAL), the second assessment of the state of adult education in the United States. Unlike the National Adult Literacy Survey (NALS) (National Center for Education Statistics, 1992), the 2003 report included 28 health literacy questions that demonstrated the lack of understanding of health information and services among U.S. adults at all literacy levels. Creating an almost “perfect storm” of curiosity, relevant literacy data, a physician champion, and a willing statewide adult and family literacy coalition, the necessary elements were in place in Wisconsin that led to the creation of a health literacy coalition. This article describes key activities and best practices that resulted in the development of WHL, a division of Wisconsin Literacy Incorporated, which has been responsible for major health literacy practices across Wisconsin during the past 15 years.

## Brief Description of Activity

The story of WHL began in 2003 when a family physician joined the board of directors of Wisconsin Literacy Incorporated. A family physician for 20 years, he had never heard anything about either the large number of adults in the United States with significant reading difficulty or the concept of health literacy. An Internet search on literacy in Wisconsin led the physician to Wisconsin Literacy Incorporated. As a member of the board of directors, he began to champion literacy and health literacy as causes deserving additional attention by a broad range of health and education organizations. Using his position as a physician and board member, he began delivering informative and persuasive health literacy presentations throughout Wisconsin and at national conferences.

It soon became clear that a lot of education was needed to raise awareness on literacy and health literacy before change or buy-in would occur within organizations. With Wisconsin Literacy Incorporated serving as a “backbone” agency, as defined in community impact models (Turner, Merchant, Kania, & Martin, 2012), there was an informal commitment by the staff and board to a three-phase approach that included (1) raising awareness and educating health care and literacy providers about health literacy, (2) implementing health literacy interventions through various grant-funded projects, and (3) disseminating project results among a broad audience of health care and adult literacy communities.

The key practices that guided the development of WHL were strategic planning retreats and an investment in a business plan to provide health literacy fee-for-service training to health care organizations. It was critical to measure the impact and outcomes of the interventions to support the claim that health literate practices, as a method to disseminate understandable health information and services, were worthy of adoption. This dissemination phase has led WHL into learning environments and communities to raise awareness and to implement health literacy pilot projects. The Midwestern states of Minnesota, Missouri, and Iowa (and later Arkansas, Texas and Kentucky) began to share information and resources on how to integrate health literacy principles, projects, and in some cases, policy, into organizational infrastructure.

What became evident in phase one of raising awareness was a lack of knowledge on the literacy levels of U.S. citizens. With each presentation delivered, a major takeaway was the shocking realization from the audience that the U.S. had an enormous literacy problem. For health care providers, there was a clear “ah-ha” moment that happened when they thought about this literacy issue in relation to patients and their challenges with access, understanding, adherence, and improved health outcomes. This literacy issue was something that health organizations needed to be aware of and begin to wrestle with its impact on quality and cost of care delivery. During this time, the national health care environment was changing dramatically. Like their counterparts across the country, Wisconsin health care organizations were facing payment reform models, and health literacy was a critical but under-recognized topic in these discussions.

Another important takeaway was that the field of adult education had knowledge and expertise in teaching reading, writing, numeracy, and language acquisition and could offer relevant approaches to health care on how adults learn best. The idea of integrating the two fields of adult education and health care seemed overwhelming in scope and unrealistic in practice. How could a statewide adult and family literacy coalition engage Wisconsin health care providers and health care organizations of all types and sizes in health literate practices? How could it be conveyed that adopting health literacy principles and activities would not be an added burden but an integrated approach to quality care embedded within their organizational infrastructure? Patient safety, patient satisfaction, hospital readmissions, medication errors, increased costs, and unnecessary emergency department visits were all topics that became part of the engagement process as there are existing issues among health care providers and ones that health literacy addresses.

## Implementation

### Raising Awareness

In 2004, Wisconsin Literacy Incorporated planned and delivered its first Health Literacy Summit with keynote speaker, Dr. David Kindig, Director of the Wisconsin Network for Health Policy Research at the University of Wisconsin and one of the editors of the Institute of Medicine's *A Prescription to End Confusion* (Kindig et al., 2004). This 1-day summit was the first Wisconsin Literacy Incorporated-hosted event focused on health literacy and was attended by approximately 50 adult literacy providers and health care professionals. This event led to more presentations by Wisconsin Literacy Incorporated's physician champion, including one key presentation to the Wisconsin Hospital Association in 2006 that was webcasted to all member hospitals. This webcast spurred additional requests from hospitals and other health care organizations looking to address patient-centered care and cost containment.

The Health Literacy Summit became a key best practice and activity of Wisconsin Literacy Incorporated in 2007 and every year thereafter, growing in content, national scope, attendance, quality, and outcomes. As the number of state and national partners increased, the level of monetary and administrative support for subsequent summits also increased. Specifically, the 2007 Summit was set up to create a sustainable coalition of health and education providers in four regions of the state, led by four regional literacy consultants employed by Wisconsin Literacy Incorporated. During the 2007 Summit, the afternoon session consisted of four meetings led by each regional literacy consultant to determine interest and commitment from attendees for starting four regional health literacy committees dedicated to bringing health care and adult education stakeholders together to raise awareness and implement health literacy initiatives within their sphere of influence. To facilitate this, the *Principles of Community Engagement* (U.S. Department of Health & Human Services, 2011) was instituted to launch and stabilize the four regional committees. This method of organizational development is defined as “the process of working collaboratively with groups of people who are affiliated by geographic proximity, special interests, or similar situations with respect to issues affecting their wellbeing” (U.S. Department of Health & Human Services, 2011). The efforts of these four regional health literacy committees enhanced awareness during the initial phases. Committees, which were composed of health care and adult education providers who volunteered to serve, convened every 4 months to consider how to raise health literacy awareness within their own communities and professional environments.

During the 2009 Wisconsin Summit, the first public unveiling of the *National Action Plan to Improve Health Literacy* (U.S. Department of Health & Human Services, Office of Disease Prevention and Health Promotion, 2010) drew a more regional audience outside of Wisconsin from states with new or active health literacy coalitions. The 2011 and 2013 Summits showcased both the *National Action Plan* and the *Ten Attributes of Health Literate Health Care Organizations* (Brach et al., 2012) by offering conference tracks based on the seven steps in the action plan or the 10 attributes from these key resources.

The biennial health literacy summits became a recognized venue for professional development in the burgeoning field. Presentations on implementation projects are encouraged to advance health literacy in the health care system and adult literacy organizations. This provided attendees with practical examples of interventions that they could apply to their work environment.

WHL has a unique practice regarding the method of conference evaluations. Like many conferences, attendees evaluated speakers and the overall conference both during and at the conclusion of the summits. In addition, 2 weeks after the summits, an evaluation form was emailed to all participants asking them to comment on specific takeaways from the conference and their intentions to act on what they learned. Three months after the summits, another evaluation was emailed to all participants asking them to report on what they had actually done or implemented since the summit. The evaluations provided insight into actions attendees had taken as a result of attending the summit.

### Instituting Health Literacy Interventions

With regional and community engagement under way, small pilot projects were started in different areas of the state. This was the second phase of work needed to build the foundation of what would eventually become WHL. The approach was to start with small projects that did not require a lot of funding but demonstrated measurable outcomes for evaluation and reporting to funders and stakeholders. After successful outcomes were achieved, it was possible to seek support for larger, multiyear projects that required more funding. Officials with Wisconsin's Department of Health Services (DHS), responsible for the state's 2010 health plan, invited Wisconsin Literacy Incorporated to give advice on the role of health literacy within the plan. Health literacy became 1 of 23 Focus Area Strategic Teams (Wisconsin Department of Health Services, 2015) for the *Healthiest Wisconsin 2020* state health plan. This plan provided a menu for action by many partners representing various sectors of health and community interest. Leveraging relationships with key staff at DHS, WHL played a strategic role in introducing the state's BadgerCare Plus health care program by providing advice on health literate ways to share this information with eligible participants.

The first part-time Health Literacy Coordinator began in 2010 as a result of an increasing number of small grant-funded health literacy projects in community literacy organizations. With a newly developed website in place and greater visibility, relationships with statewide health care organizations such as Wisconsin Medical Society, Pharmacy Society of Wisconsin, and Wisconsin Health Information Organization were formed. The amount of work coming from the state Wisconsin Literacy Incorporated office under the direction of the new health literacy coordinator increased rapidly. With all the work dedicated solely to health literacy advancement, it seemed appropriate to launch a separate division, first titled Health Literacy Wisconsin and later renamed Wisconsin Health Literacy.

For project implementation, WHL used a key best practice of delivering health information in ways that people with low health literacy were able to understand. The division designed workshops to increase knowledge-based health information and services in trusted community settings where people regularly went for services or community activities. Workshop curriculum were developed on a wide variety of topics such as what to do for sick or injured children, maternal health, proper use of insurance, influenza and vaccination, and prescription medication use and safety. These pilot projects were designed to be replicable, scalable, sustainable, and delivered at 4th- to 6th-grade reading levels to achieve better understanding by participants.

### Disseminating Health Literacy Practice and Projects with Partners

After several years of implementation projects, WHL began a third phase of building its health literacy division that involved sharing and disseminating health literacy practices and projects with partners from within and outside Wisconsin. With other national health literacy activities came efforts in various states to build health literacy coalitions or health literate organizations. Communication among partners in different states began at regular intervals to share best practices, resources, and ideas on building sustainable coalitions. In the Midwest, the states of Minnesota, Iowa, Missouri, and Wisconsin formed the Health Literacy Regional Network (HLRN) in 2011. Aided by their close proximity, this group scheduled quarterly calls and several in-person visits to Wisconsin and Missouri to learn from each other. Soon, Arkansas and Texas joined to learn how to support health literacy work within their states. States such as New Jersey, Pennsylvania, Colorado, and Kentucky hosted inaugural health literacy conferences, drawing on support from active health literacy coalitions or organizations in other states. States began sharing how they were building coalition infrastructure, launching fee-for-service training, hosting conferences, and building new partnerships.

The national Health Literacy Discussion list supported by the Institute for Healthcare Advancement was another effective method for disseminating information. The moderated discussion list provides a professional Community of Practice (CoP) that fosters the exchange of information, building on the health literacy knowledge and skills of the wide range of health and education professionals contributing to the online discussions (E. Wenger-Trayner, & Wenger-Trayner, 2011). This best practice provided the most consistent and frequent forum for learning, exchanging ideas, sharing resources, engaging in discourse on current issues in the field, promoting activities, and building relationships that transcended geographic boundaries and connected professionals across a diverse range of health and education professions. This CoP allowed WHL to share its work both nationally and internationally, and also provided an opportunity for WHL members to be guest discussion leaders on several occasions and share their experiences of health literacy work with a broad audience.

## Results

During a 15-year period from 2003 through 2018, the best practices of raising awareness, implementing pilot projects, and participating in communities of practice helped develop, grow, and sustain a health literacy coalition and division within a statewide adult literacy coalition. Identifying a health literacy champion was an initial and critical best practice that allowed Wisconsin Literacy Incorporated to consider its role in supporting efforts to raise awareness among key health care and adult education stakeholders. Two strategic planning retreats in 2008 and 2010 guided the work of the regional health literacy committees, and a business plan developed in 2013 guided how the WHL division would sustain a fee-for-service model for health literacy training in health care organizations.

### Regional Health Literacy Committees

Spending a minimum of 6 years intentionally raising awareness among key health care and education venues both in and outside of Wisconsin also contributed to Wisconsin Literacy Incorporated's ability to model Principles of Community Engagement. This supported four regional health literacy committees within Wisconsin that raised health literacy awareness, implemented projects, secured organizational buy-in from health care leadership, and hosted regional conferences. Organized by Wisconsin Literacy Incorporated regional staff but comprised by health care and education providers who served as volunteers, these regional committees became difficult to sustain as more health literacy work was being done by the Wisconsin Literacy Incorporated office in 2010, soon after the first health literacy coordinator was hired. Volunteer committee members with their own time constraints and professional obligations found it increasingly difficult to volunteer the time for health literacy activities within their respective regions. By 2013, these four regional committees dissolved, and an increasing amount of work focused on small implementation projects directed from the newly launched WHL division. This resulted in a transition from a statewide voluntary health literacy coalition to a health literacy division within a statewide adult literacy coalition.

### Summits

The cumulative results of hosting seven biennial WHL Summits on the formation and sustainability of the WHL are significant. Although such events were part of the raising awareness work of the division, they also provided formative professional development opportunities for attendees and WHL staff, and improved the division's ability to share current health literacy tools, research, implementation projects, and best practices with a broad range of health and education providers. Most of the attendees were from Wisconsin, but the Summits continued to draw a national and international group of breakout presenters, plenary speakers, and attendees from 31 states and 3 international countries. Evaluation questionnaires completed by participants 3 months after attending summits listed a variety of results such as new coalitions being formed, providers communicating with patients using health literate tools, and receiving buy-in from executive leadership to implement health literate practices.

### Implementation Projects

Guided by the planning retreat outcomes and a business plan to support the new division, WHL staff successfully obtained funding to implement pilot projects in the community and with health care organizations. Targeted health care organizations included those with statewide reach, such as the Wisconsin Medical Society, Wisconsin Hospital Association, Wisconsin Academy of Family Physicians, Wisconsin Nurses Association, and Wisconsin Department of Health Services. Projects were chosen on their ability to affect adults (primarily those in local member literacy agencies) challenged by finding, understanding, and acting on health information and services. Projects also were selected by the opportunity to replicate, scale, and sustain the project. As a result, WHL developed and disseminated several statewide pilot projects that focused on increasing health knowledge around specific health issues. For example, in 2010, a project to address H1N1 influenza, funded by the Centers for Disease Control and Prevention through the Wisconsin Department of Health Services Minority Health division, allowed WHL to offer “Let's Talk About the Flu” community workshops (https://wisconsinliteracy.org/health-literacy/what-we-do/lets-talk-about-the-flu.html). Later, this project attracted private funding from the Anthem Blue Cross and Blue Shield Foundation to increase the number and impact of the workshops statewide and to measure increased knowledge and flu vaccination rates. Of those attending the workshops, 55% reported getting a flu shot, compared with an average of 32% of all Wisconsin adults receiving the shot. This project also was adopted by Arkansas's State Department of Health in 2015. Through shared best practices and a training webinar, WHL was able to share this project with Arkansas' Immunization Action Coalition, which developed its own “Let's Talk About the Flu” statewide effort.

Literacy agency directors, who follow up with WHL staff after hosting a workshop, offered a strong validation of the positive impact of the intervention on literacy students' learning. One director said:

Individuals living in poverty are frequently unable to purchase nutritious food, obtain preventive health care and screenings, and/or afford the costs of medication. They may also have difficulty accessing and/or understanding health information or reading medication labels. The WHL workshops presented information on timely and relevant topics in ways that were useful and applicable to our clients (i.e., appropriate reading level, web-based, resource materials, facilitated workshops). Clients came to Jefferson County Literacy Council through word-of-mouth advertising by satisfied customers. Learners stayed if they felt they were receiving high-quality, value-added services. The WHL workshops gave us an opportunity to offer services above and beyond the regular ABE (adult basic education) or ESL (English as a second language) curriculum. The workshops were on topics of interest to clients and they were well developed and presented. Therefore, they also served as a marketing and recruitment tool for our agency.

Another director of an urban program was asked, “How do you see the workshops as affecting the overall literacy development of your students?” The director said:

Not only were the materials provided simple and easy to follow, but they offered ample visual aids to help my ELL (English language learner) students make connections between the pictures and the words on the page. Going through a prescription label, for example, demystified the sea of information that one can find on any given prescription label. Students got exposure to and practice with numeracy and measurements, identifying dosages and interpreting instructions on how much and how often to take a medicine by reading the label closely.

Another rural agency director said:

Students have had the opportunity to participate in workshops on various topics such as reading medication labels, purchasing health insurance, and understanding pain medications. Students had the opportunity to discuss their own experiences and to learn new information from the facilitator and the materials presented. As evidenced by pre- and posttests, all participants felt they learned new information. In addition to acquiring content knowledge of the subject matter, students received opportunities to read the materials, participate in group discussions, formulate and ask critical questions, and apply information to their daily lives

(Wisconsin Health Literacy, 2018).

### Dissemination

The WHL's efforts in the third phase of development, sharing with other CoP, took place at regular intervals as WHL hosted HLRN quarterly calls with other state health literacy personnel and advocates. States especially learned from each other as fee-for-service efforts in health literacy training were offered. The group discussed approaches on fee structures, marketing, and service delivery. More recently, such CoP offered shared experience on policy issues in health literacy as Kentucky faced proposed legislation changes requiring Medicaid beneficiaries to take an online health literacy assessment before their benefits would be granted. This topic was an agenda item for the HLRN call in April 2018 and also received attention from the Institute for Healthcare Advancement (IHA) discussion listserv. Regardless of the platform, WHL has benefited from and provided benefits for health literacy CoP, including those in person, through teleconferencing and online.

## Lessons Learned

Building the capacity of a statewide adult literacy coalition to launch and support a health literacy division under its umbrella provided many opportunities in Wisconsin and beyond to positively impact the understanding and commitment to improved health literacy. Best practices allowed for the division's creation, recognition among key health care stakeholders, implementation of health literacy projects, and dissemination in broader CoP. Under the three main areas of work (raising awareness, implementing pilot projects, and disseminating information and best practices), WHL has positively affected both the field of health literacy and adult basic education.

Important lessons learned included benefits of working with a champion to open doors and influence conversations in health care settings that were not available to Wisconsin Literacy Incorporated staff early on in the division's development. Dedicating a minimum of 6 to 7 years to intentionally raise awareness in strategic health care settings provided a solid foundation and numerous partnerships with health care stakeholders. The WHL invested in building strong relationships over time among stakeholders representing a multiplicity of voices. The simultaneous development of both a strategic plan and a business plan helped periodically assess and evaluate current and past accomplishments, as well as future directions. The planning also facilitated the work of volunteer regional health literacy committees transferring to the state office, which served as the hub for regional and statewide health literacy work. It also allowed the new WHL division to plan sustainability efforts from various revenue streams.

For implementation of pilot projects and community interventions, WHL began with small manageable projects that could be replicated and scaled statewide. This resulted in WHL developing ongoing relationships with funders, building on previous success, and obtaining funding from both new and previous sources to continue project work. A difficult lesson was learning to not take on too much project activity at once and being intentional about what aligned with the organization's mission and capacity.

Working with adult learners who were advisors to health care and to WHL became an important part of delivering effective health information at an accessible and understandable level. Connecting with adult learners in small focus groups to inform pilot projects allowed WHL to design materials with learner goals in mind. Another important best practice was delivering educational workshops in community-based organizations and trusted settings where adults regularly go to learn or engage in community. WHL staff learned from local literacy providers. One staff member said:

Students loved the workshops! Students have come back to class and reported things like moving their medicines out of the bathroom, getting measuring instruments from the pharmacy, and using their pill boxes from the workshop to organize their medicines. I think the fact that students went home and immediately started implementing what they learned says so much about how the workshops affected their understanding of health information.

In the third phase of the project, dissemination of best practices and engaging in CoP, WHL developed important relationships with key partners inside and outside of the state. This enhanced program development and execution by sharing project components with other health and education partners. The most impactful CoP initially was the Health Literacy Regional Network in which regular meetings and some in-person visits allowed for more ground-level sharing of health literacy interventions. The IHA Health Literacy Discussion Listserv became a critical venue to disseminate how the division established its infrastructure and how the pilot and intervention projects were developed and delivered in community settings. In addition, the seven previous WHL summits played a big role in providing a national venue to disseminate the division's work and that of the field.

WHL, as a division of Wisconsin Literacy Incorporated, has a bright future. Given best practices executed during its development, the prospects for sustaining its work and impact are promising. All three areas of its development—raising awareness, implementing health literacy interventions, and disseminating best practices in communities of practice —will need continual attention and support. Next steps include following strategic plan activities focusing on building and maintaining a strong and stable infrastructure, seeking grant revenue for community outreach, and marketing fee-for-service training and services. These activities will not only foster sustainable growth within the health literacy division but also will positively impact its parent organization, Wisconsin Literacy Incorporated, and the field of adult basic education.
